# Comparison of multi echo T_2_ relaxation and steady state approaches for myelin imaging in the central nervous system

**DOI:** 10.1038/s41598-020-80585-7

**Published:** 2021-01-14

**Authors:** Adam V. Dvorak, Emil Ljungberg, Irene M. Vavasour, Lisa Eunyoung Lee, Shawna Abel, David K. B. Li, Anthony Traboulsee, Alex L. MacKay, Shannon H. Kolind

**Affiliations:** 1 Physics and Astronomy, University of British Columbia, Vancouver, BC Canada; 2 International Collaboration on Repair Discoveries (ICORD), University of British Columbia, Vancouver, BC Canada; 3 Department of Neuroimaging, Institute of Psychiatry, Psychology & Neuroscience, King’s College London, London, UK; 4 Radiology, University of British Columbia, Vancouver, BC Canada; 5 Medicine (Neurology), University of British Columbia, Vancouver, BC Canada

**Keywords:** Magnetic resonance imaging, Central nervous system, Biomarkers, Myelin biology and repair, Multiple sclerosis

## Abstract

The traditional approach for measuring myelin-associated water with quantitative magnetic resonance imaging (MRI) uses multi-echo T_2_ relaxation data to calculate the myelin water fraction (MWF). A fundamentally different approach, abbreviated “mcDESPOT”, uses a more efficient steady-state acquisition to generate an equivalent metric (f_M_). Although previous studies have demonstrated inherent instability and bias in the complex mcDESPOT analysis procedure, f_M_ has often been used as a surrogate for MWF. We produced and compared multivariate atlases of MWF and f_M_ in healthy human brain and cervical spinal cord (available online) and compared their ability to detect multiple sclerosis pathology. A significant bias was found in all regions (p < 10^–5^), albeit reversed for spinal cord (f_M_-MWF =  − 3.4%) compared to brain (+ 6.2%). MWF and f_M_ followed an approximately linear relationship for regions with MWF <  ~ 10%. For MWF >  ~ 10%, the relationship broke down and f_M_ no longer increased in tandem with MWF. For multiple sclerosis patients, MWF and f_M_ Z score maps showed overlapping areas of low Z score and similar trends between patients and brain regions, although those of f_M_ generally had greater spatial extent and magnitude of severity. These results will guide future choice of myelin-sensitive quantitative MRI and improve interpretation of studies using either myelin imaging approach.

## Introduction

Quantitative magnetic resonance imaging (MRI) techniques are developed with the aim of being specific to certain features of tissue microstructure. In the case of myelin water imaging, the water trapped between myelin bilayers can be used as a biomarker for myelin content.

The original method for myelin water imaging was a multi-echo spin-echo T_2_ relaxation acquisition, which can quantify signal contributions from different characteristic T_2_ relaxation times, such as those associated with water trapped between myelin lipid bilayers (T_2_ < 40 ms at 3 T) or water within intra- and extra-cellular spaces (40 ms < T_2_ < 200 ms)^1^. The myelin water fraction (MWF) metric can then be calculated as the signal contribution from T_2_ relaxation times associated with myelin water divided by the total signal. Although this model neglects the presence of non-aqueous protons and the effects of magnetization exchange between water pools^2,3^, the MWF has been shown to correlate well with quantitative histopathologic measures of myelin density^4–6^. A multi-echo 3D GRAdient-echo and Spin-Echo (GRASE) sequence can be used to reduce acquisition time by a factor of 3 with a negligible reduction in the resulting MWF map quality compared to a multi-echo spin-echo sequence^7,8^.

The value of an in-vivo myelin biomarker has motivated the development of alternative approaches to myelin imaging. One commonly used alternative is the multi-component Driven-Equilibrium Single-Pulse Observation of T_1_ and T_2_ (mcDESPOT) technique, which is based on a series of steady-state acquisitions^9^. Spoiled gradient recalled echo (SPGR), inversion-recovery-prepared SPGR (IRSPGR) and balanced steady-state free precession (bSSFP) images are acquired with a range of flip angles (FA), from which T_1_ and T_2_ can be estimated. The mcDESPOT analysis framework requires fitting the data to a large number of parameters simultaneously, including separate, exchanging T_1_ and T_2_ components for myelin-associated and intra-/extra-cellular water, which facilitates calculation of the mcDESPOT myelin water fraction (f_M_). Compared to myelin imaging with multi-echo T_2_ relaxation, mcDESPOT is appealing for its intrinsic acquisition efficiency^10^, which can be leveraged to improve coverage, resolution, or acquisition time, and for its additional quantitative metrics, such as T_1_ and exchange rate constants, which can provide supplemental information.

Potential inaccuracies or biases of the GRASE and mcDESPOT analysis procedures have previously been explored. Multi-component T_2_ analysis of GRASE, or any multi-echo T_2_ relaxation data, relies on suitable analysis parameters and sufficient data signal-to-noise ratio for MWF values to stabilize^11^. mcDESPOT analysis requires accurate parameter fitting boundaries to be defined, but even then has been criticized for non-unique solutions and inherent, unpredictable bias in the fitting procedure^12–15^.

Similarly, both acquisitions have a set of MR-signal related potential confounds for myelin imaging, which have been reasonably well studied^16–24^. Previous studies have found differences between MWF and f_M_ in brain tissue, with mcDESPOT providing consistently higher myelin estimates, especially in gray matter (GM), and a much more uniform distribution of values, especially in white matter (WM)^24–26^. However, MWF and f_M_ values reported in cervical spinal cord are relatively similar^27,28^, which is surprising given their disparity in the brain. This difference between brain and spinal cord could be related to the influence of MR physics related confounding factors, bias in analysis procedures, differences in cyto-architecture between brain and spinal cord, or likely a combination of the above.

Despite these differences, both GRASE and mcDESPOT have individually demonstrated sensitivity to pathology and developmental changes in the central nervous system, often with conclusions that corroborate one another^26,29–32^. Previous work has reported a significant, moderately strong correlation between MWF and f_M_ in brain tissue^26^, further suggesting that steady-state approaches can serve as effective alternatives to multi-echo T_2_ relaxation myelin imaging.

However, as previously reported values suggest that this relationship might not hold in the spinal cord, a direct comparison with spinal cord data would be of great value as it could strengthen or contradict the purported correspondence between MWF and f_M_.

In this study, we perform the first direct comparison of multi-echo T_2_ relaxation (GRASE) and steady-state (mcDESPOT) myelin imaging in both the brain and cervical spinal cord in a cohort of healthy participants. Our comparison is supplemented with data in the context of demyelinating disease pathology, analyzed on an individual basis for people with multiple sclerosis. The goals of this study are to:

- create atlases of normative reference MWF and f_M_ values for both brain and cervical spinal cord;
- use atlas-based methods to compare myelin imaging approaches with minimal influence from biological variation and measurement noise;
- improve characterisation of the relationship between MWF and f_M_, by comparing values across a diverse range of tissue, including healthy and diseased brain and spinal cord.

## Results

Five of the 28 participants had to be excluded from the MWF and f_M_ spinal cord atlases, due to imaging artefacts that rendered their GRASE or mcDESPOT data unusable.

### Mean atlas ROI values

Table 1 provides the mean and standard deviation (SD) of voxel values within each brain and spinal cord region of interest (ROI), calculated for the mean MWF and f_M_ atlases. Mean MWF and f_M_ ROI values across all healthy control participants differed significantly (p < 10^–5^) for all ROIs. f_M_ values were higher than MWF in all brain ROIs by a factor of ~ 2, except for the posterior internal capsule where values were more similar (f_M_ = 19.5 ± 1.2%, MWF = 17.6 ± 4.0%) and the caudate where f_M_ was larger by a factor of ~ 3 (f_M_ = 10.0 ± 3.3%, MWF = 3.0 ± 1.8%). In spinal cord, f_M_ was lower than MWF in all ROIs except GM and the absolute difference between f_M_ and MWF values was smaller than in brain.

**Table 1:** Mean and standard deviation (SD) of voxels within each region of interest (ROI) for the mean myelin water fraction atlases from multi-echo T_2_ relaxation (MWF) and steady-state (f_M_) approaches.

ROI	MWF (*%*)	f_M_ (*%*)	ROI volume
	Mean ± SD	Mean ± SD	Number of voxels
All WM&GM	5.7 ± 5.4	11.9 ± 5.5	1,239,957
**Brain white matter**			
All WM	8.0 ± 4.9	16.8 ± 4.1	538,746
All JHU	11.5 ± 4.5	19.9 ± 2.7	78,903
Genu	9.1 ± 2.3	20.0 ± 3.0	5260
Splenium	13.4 ± 2.8	20.4 ± 3.1	7527
CC	11.1 ± 3.0	19.6 ± 3.0	21,435
Posterior internal capsule	17.6 ± 4.0	19.5 ± 1.2	5437
**Brain lobe white matter**			
Frontal	7.1 ± 3.5	17.6 ± 3.4	115,698
Occipital	7.3 ± 3.2	16.0 ± 3.9	72,205
Parietal	8.3 ± 3.7	17.8 ± 3.6	100,925
Temporal	6.8 ± 2.9	16.4 ± 3.7	45,173
**Brain gray matter**			
All GM	4.1 ± 5.2	8.1 ± 2.7	701,211
Cortical	3.7 ± 4.1	8.7 ± 2.8	445,356
Caudate	3.0 ± 1.8	10.0 ± 3.3	8130
Thalamus	7.4 ± 5.2	13.2 ± 3.7	14,105
Putamen	5.0 ± 3.8	12.0 ± 2.6	13,748
**Spinal cord**			
Whole	22.1 ± 5.7	18.7 ± 2.9	18,673
All WM	23.8 ± 4.4	18.6 ± 3.2	14,614
All GM	15.7 ± 5.1	19.0 ± 1.0	4059
Dorsal column	25.6 ± 4.6	19.2 ± 2.5	5541
Lateral funiculi	22.8 ± 3.9	18.3 ± 3.3	7235

ROI volumes are also provided. A paired, two-tailed t test found significantly different MWF and f_M_ ROI values (p < 10^–5^) across healthy control participants, for all ROIs. Brain ROIs include all white matter (WM), all gray matter (GM) and combined WM&GM from T_1_w image segmentations, along with 9 additional WM ROIs (all JHU white matter labels combined, genu of corpus callosum, splenium of corpus callosum, whole corpus callosum (CC), posterior internal capsule, and frontal, occipital, parietal, and temporal lobes masked to WM), and 4 additional GM ROIs (cortical GM, caudate, thalamus, and putamen). Spinal cord ROIs include the whole cord, WM, GM, dorsal column, and lateral funiculi.

### Healthy participant ROI values

**Figure 1:**
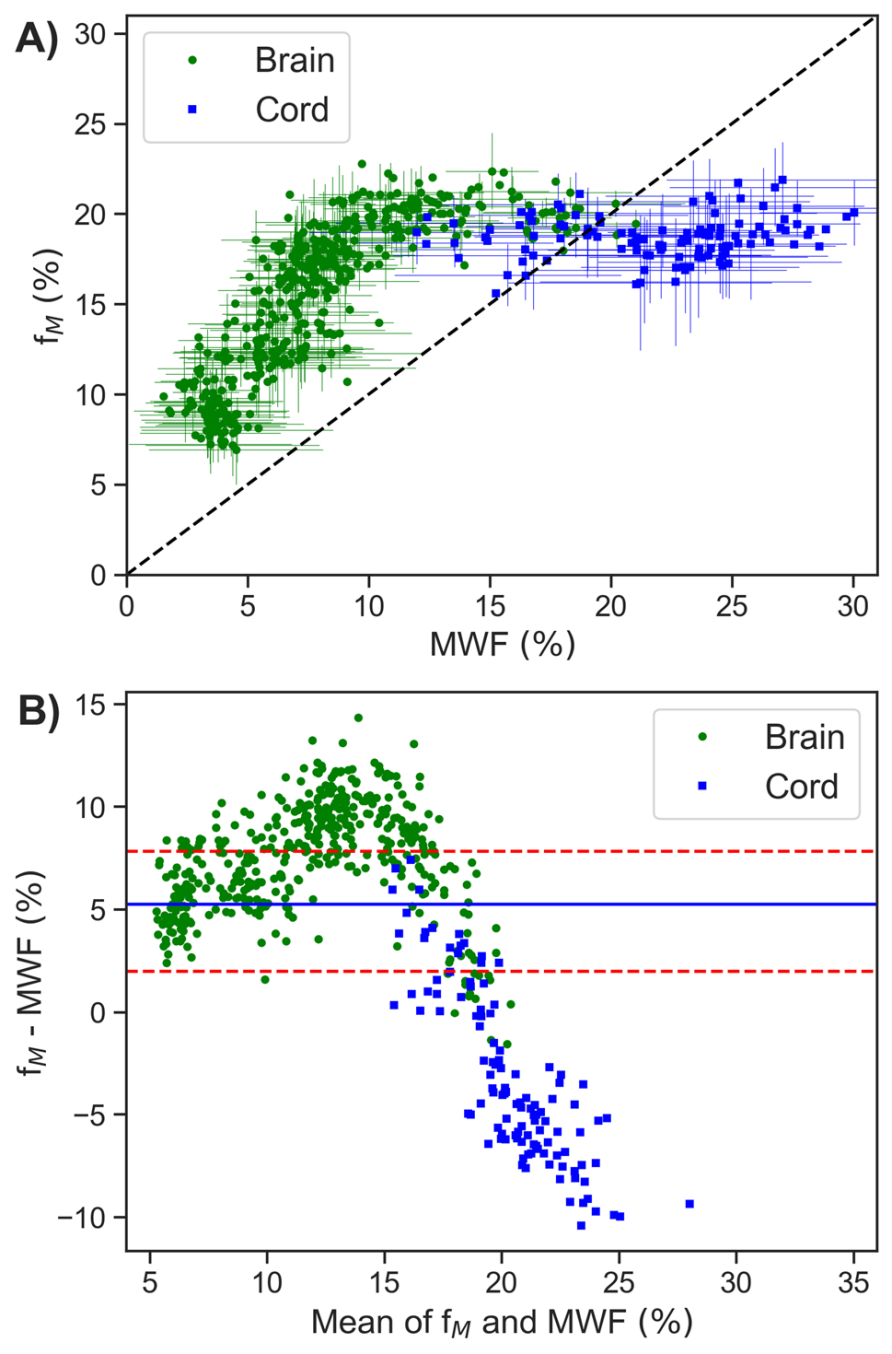
Mean myelin water fraction values for each region of interest (ROI), for each healthy control, from multi-echo T_2_ relaxation (MWF) and steady-state (f_M_) approaches. (**A**) A correlation plot between MWF and f_M_, where the dotted line indicates the line of unity. Linear regression was omitted, due to the clearly non-linear relationship between MWF and f_M_. Uncertainty bars indicate the standard deviation of voxel values for that subject and ROI, only plotted for every third data point to improve visibility. (**B**) Bland Altman plot displaying the mean of MWF and f_M_ values on the x axis and the difference on the y axis. The solid line indicates the mean bias (difference) of all data points (+ 5.3%). Dotted lines indicate positive and negative 95% limits of agreement; + 7.8% and + 2.0%, respectively. ROIs include all white matter (WM), all gray matter (GM) and combined WM&GM from T_1_w image segmentations, along with 9 additional WM ROIs (all JHU white matter labels combined, genu of corpus callosum, splenium of corpus callosum, whole corpus callosum, posterior internal capsule, and frontal, occipital, parietal, and temporal lobes masked to WM), and 4 additional GM ROIs (cortical GM, caudate, thalamus, and putamen). Spinal cord ROIs include the whole cord, WM, GM, dorsal column, and lateral funiculi.

In Fig. 1 mean MWF and f_M_ values within each ROI, for each healthy participant, are displayed. Figure 1A shows f_M_ plotted against MWF, for each ROI value. The Bland–Altman plot in Fig. 1B shows the difference between each MWF and f_M_ ROI value plotted against their mean.

There was an approximately linear relationship between MWF and f_M_ for brain regions (circle data points in Fig. 1A) with MWF <  ~ 10%. The linear MWF-f_M_ relationship did not hold in the spinal cord, or in brain regions with MWF >  ~ 10%. The non-linearity between f_M_ and MWF appears qualitatively consistent across participants, with a tight spread of data points following the same distinct pattern. The intra-subject, intra-ROI variability of each metric, indicated by standard deviation uncertainty bars in Fig. 1A, is small compared to the range of f_M_ and MWF values and could not account for the non-linear relationship.

In the spinal cord, MWF spanned a large range of values (12–30%) whereas f_M_ had little variation (16–23%). A systematic bias exists between MWF and f_M_ values, but with opposite sign for brain (mean WM&GM f_M_-MWF = 6.2%) compared to spinal cord (mean whole cord f_M_-MWF =  − 3.4%).

### Templates and atlases

**Figure 2:**
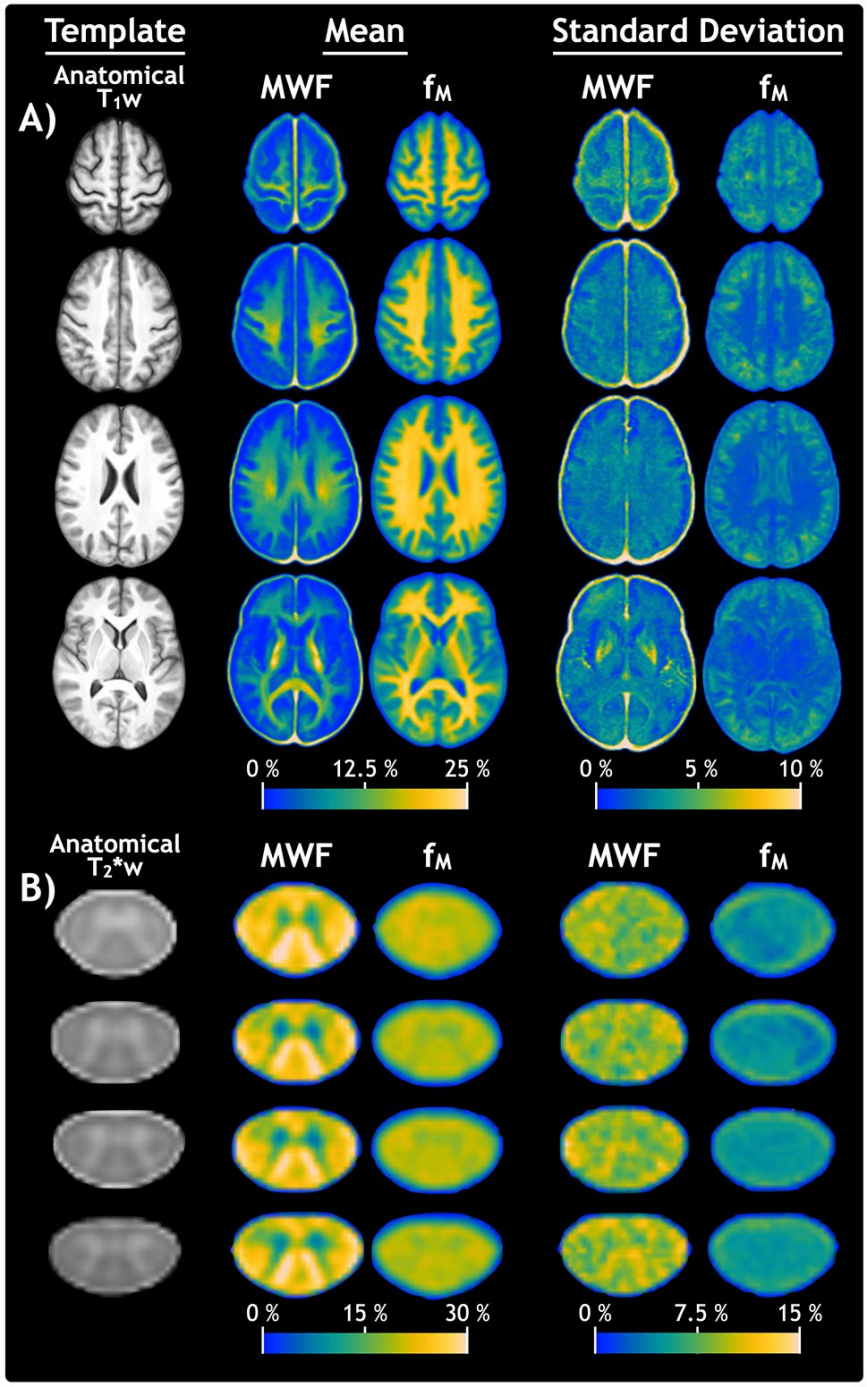
Representative slices of the anatomical templates as well as quantitative atlases for the myelin water fraction from multi-echo T_2_ relaxation (MWF) and steady-state (f_M_) approaches, for the (**A**) brain and (**B**) C2 and C3 level of the cervical spinal cord. From left to right, columns display the: anatomical template, mean atlases, and the standard deviation atlases. Mean and standard deviation atlases are calculated voxel-wise across 28 participants for brain and 23 participants for spinal cord.

Representative slices of the anatomical templates, T_1_-weighted (T_1_w) for brain and T_2_*-weighted (T_2_*w) for spinal cord, voxel-wise mean MWF and f_M_ atlases, and voxel-wise SD MWF and f_M_ atlases are displayed in Fig. 2A for brain and Fig. 2B for spinal cord. As observed in Fig. 1A, there is a larger dynamic range in the myelin estimates with MWF compared to f_M_. In the mean MWF atlas of the spinal cord, structures such as gray matter and dorsal column can be identified clearly. The templates, mean and SD atlases, and ROIs analyzed for MWF and f_M_ in brain and spinal cord are openly available online^33^.

### Z scores

**Figure 3:**
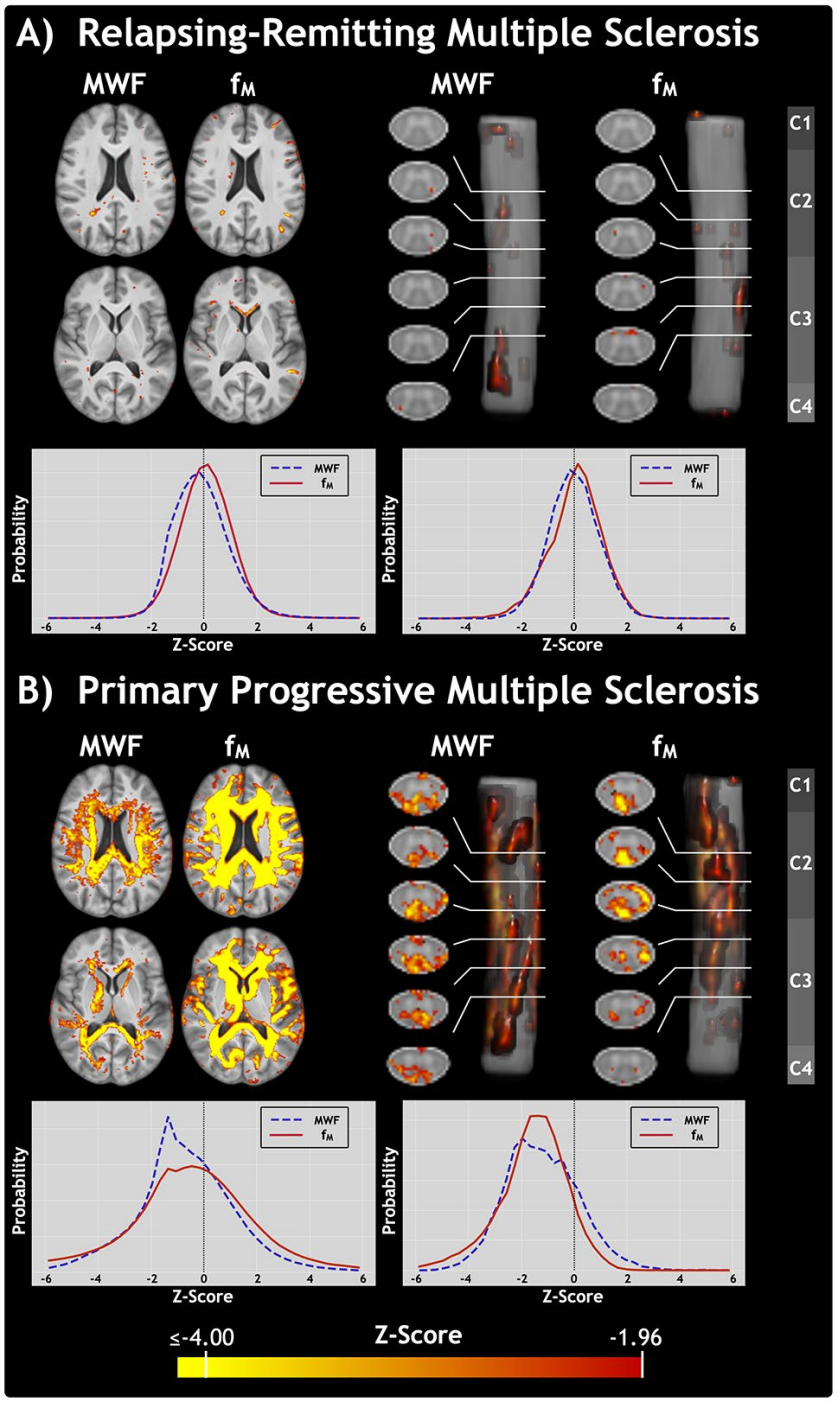
Representative slices of Z score maps for the myelin water fraction from multi-echo T_2_ relaxation (MWF) and steady-state (f_M_) approaches. Deficient Z scores, with Z ≤  − 1.96, are superimposed on the anatomical templates for (**A**) one participant living with relapsing–remitting multiple sclerosis (female, age 26 years, expanded disability status scale (EDSS) 1.5, disease duration 3 years) and (**B**) one with primary progressive multiple sclerosis (female, age 64 years, EDSS 8.5, disease duration 29 years). From left to right, columns display: axial brain slices, axial cervical spinal cord slices, sagittal cervical cord views, and the corresponding vertebral levels of the cord. For brain and cord of each patient, histograms are plotted to show the distribution of Z score values for MWF (dashed blue line) and f_M_ (solid red line). Z scores were calculated as the difference between multiple sclerosis metric values and the mean atlas, divided by the standard deviation atlas.

Representative slices of Z score maps comparing relapsing–remitting multiple sclerosis (RRMS) and primary progressive multiple sclerosis (PPMS) MWF and f_M_ to the healthy participant atlases are shown in Fig. 3 for brain and for spinal cord. The RRMS Z score maps, Fig. 3A, show evidence of some small, focal areas of deficient MWF and f_M_ compared to controls, likely corresponding to the location of lesions. The PPMS Z score maps, Fig. 3B, show widespread regions of low Z score for both MWF and f_M_ in both brain and spinal cord.

In Fig. 3, histograms for brain and cord of each patient show similar distributions of Z score values for MWF and f_M_, with the largest differences visible in the extremes of the Z score range for PPMS. For RRMS, a slight shift towards lower values is apparent for the MWF Z score histogram, compared to f_M_. For PPMS, the MWF Z score maps are generally less extreme in terms of both spatial extent and statistical magnitude compared to those of f_M_. While PPMS Z score maps suggest that f_M_ has greater sensitivity to the presence of pathological tissue changes, RRMS Z score histograms suggest that MWF may better identify subtle changes outside regions of more-extreme pathology. Regions of deficient MWF Z score are generally highlighted on the corresponding f_M_ Z score map, although this congruity appears to be more consistent in brain.

## Discussion

The atlas-based methods employed here provided an effective means of reducing biological variations and measurement noise present within and between individual participant quantitative metric maps. In addition to facilitating the creation of Z score maps, atlas-based methods provide increased statistical power for characterizing discrepancies between the quantitative myelin fraction metrics obtained with GRASE and mcDESPOT. The smooth, “high SNR” appearance of the atlases in Fig. 2 provide clear depictions of representative values expected from each imaging modality. They can also serve as a reference for how MWF and f_M_ values are expected to differ throughout commonly investigated regions in the central nervous system. The MWF and f_M_ atlases are openly available and provide normative reference data to increase statistical power for future studies with limited ability to recruit and collect data from healthy controls.

Although previous studies have compared MWF and f_M_ in the brain, this is the first comparison of MWF and f_M_ values in both the brain and spinal cord. Our combined analysis yielded some consequential results, especially when interpreting spinal cord results in concert with those from brain. First, the absolute difference in magnitude between myelin-related metric values produced by multi-echo T_2_ relaxation and steady-state approaches is much smaller in spinal cord than in brain. Despite having more-similar magnitudes than in brain, MWF and f_M_ in the spinal cord bear almost no correspondence. Secondly, the average bias between GRASE and mcDESPOT is reversed in spinal cord (mean whole cord f_M_-MWF = − 3.4%) compared to brain (mean WM&GM f_M_-MWF = 6.2%). The inversion of the MWF versus f_M_ bias between brain and spinal cord is clearly depicted by the individual participant mean ROI values plotted in Fig. 1A, where most brain and spinal cord data points lie on opposite sides of the line of unity.

Although comparisons in human brain have shown significant, moderately strong correlations between MWF and f_M_^26^, the same linear relationship does not hold in the spinal cord. Figure 1A suggests that this relationship actually begins to break down in brain regions as well. Specifically, MWF and f_M_ do not appear to be correlated in regions with MWF >  ~ 10%, where f_M_ no longer increases in tandem with MWF. In hindsight, this is visible in some brain regions examined by previous studies^26,34^, but the magnitude and extent of the incongruity between the two methods is made clear by our unified, direct comparison with both brain and spinal cord data.

Our atlas ROI results (Table 1) show that MWF is lower than f_M_ throughout all investigated brain ROIs, in agreement with previous studies that compared values in the brain^24–26^. Lack of contrast in f_M_ between WM ROIs, as demonstrated by Table 1 values and qualitatively by mean f_M_ atlases in Fig. 2, also matches expectations based on previous brain studies with mcDESPOT^24–26^.

Previous work has provided evidence that myelin content within human brain WM is heterogeneous. Aboitiz et al. studied the corpus callosum in post-mortem human brain using the Loyez histological stain for myelin and electron microscopy^35^. They found that the genu had a smaller median axon diameter (0.6 μm) compared to the splenium (1.0 μm), which is expected to correspond with lower oligodendrocyte proliferation and myelin content^35^. They also found a high percentage of unmyelinated fibers in the genu (16%) compared to the rest of the corpus callosum (< 5%)^35^. Both of these findings support the expectation of lower myelin content in the genu, compared to the splenium, which we found reflected in our results for MWF but not for f_M_. In more recent studies using histological staining markers for myelin, the optical density of Luxol fast blue showed regional variation by factors > 2 within non-lesional WM, albeit in MS brain tissue where pathological changes could increase stain contrast^5,6^. Bürgel et al. demonstrated strong regional contrast in WM myelination when they used a modified Heidenhain–Woelcke stain^36^ to distinguish major tracts (including the corticospinal tract, optic and acoustic radiations, fornix, cingulum, corpus callosum, superior longitudinal, superior and inferior occipito-frontal and uncinate fascicles) in 10 post-mortem brains by their relative degrees of myelination^37^. Therefore, the low contrast of f_M_ within WM is likely not representative of myelin content but rather could relate to a confounding factor for mcDESPOT, such as the increased influence of magnetization transfer. Similarly to f_M_, magnetization transfer ratio maps show relatively little contrast between WM regions compared to MWF^38^, but studies investigating the relationship between magnetization transfer and mcDESPOT have not been able to confirm that magnetization transfer has a prominent influence on f_M_ values^24^.

Our GRASE and mcDESPOT values agree with mean whole cord MWF and f_M_ values reported previously by our group in healthy cervical spinal cord: 22.1% versus 22.9% reported by Ljungberg et al.^27^ and 18.7% versus 20.5% reported by Kolind et al.^28^. In our study, WM accounted for 78% of the spinal cord volume, compared to 43% of brain tissue volume. As a result, higher mean MWF and f_M_ can generally be expected across the whole spinal cord. Although this does not explain higher MWF in both WM and GM individually, previous studies have reported approximately 2.5 times higher myelin content in the cord, compared to the cerebrum, of rat nervous system^39^, providing a precedent for a similar pattern to exist in human tissue.

There are many possible explanations for the low f_M_ in the spinal cord, compared to expectations from the brain. Like all quantitative MRI techniques, GRASE and mcDESPOT each have their own set of confounds, which could affect the results more or less in the spinal cord, causing the relationship between MWF and f_M_ to change.

GRASE is expected to have some magnetization transfer effects due to repeated refocusing pulses throughout the echo train^40^. For mcDESPOT, the short, high-amplitude excitation pulses used to acquire SPGR and bSSFP data minimize undesirable finite radio-frequency pulse effects but increase the effects of magnetization transfer. By sensitizing the signal to macromolecular protons present in biological tissue, magnetization transfer can have a profound effect on estimates of T_1_ and T_2_ relaxation values^41–43^. Modified versions of mcDESPOT have been introduced with additional model parameters in an attempt to quantify and correct for magnetization transfer^44^. Upon investigation, Zhang et al. confirmed that removal of magnetization transfer has a strong effect on bSSFP signal curves. However, values for f_M_ remained much higher than MWF, suggesting that magnetization transfer does not drive the MWF-f_M_ difference in brain. The fact that signal changes did not propagate into the expected parameter changes helped motivate subsequent investigation into the mcDESPOT analysis procedure.

The GRASE MWF is generally underestimated in regions with very low signal contribution from myelin water, such as cortical GM brain regions, where there is insufficient signal-to-noise for accurate myelin water quantification^45^. The multi-component T_2_ analysis used with GRASE ignores the effects of T_1_ relaxation. Using a short repetition time (TR) with GRASE could therefore artificially increase MWF values, due to additional T_1_ weighting. However, our TR of 1073 ms is not expected to have a large influence on MWF and does not explain the discrepancy with f_M_ cord values^46^. Recent studies have shown that the MWF will decrease if the inter-compartmental exchange rate between myelin water and intra/extra-cellular water increases^40,47^. Therefore, GRASE MWF values may be underestimated, especially in regions with small axons and thin myelin sheaths, where an increase in inter-compartmental exchange can be expected^48^. In comparison, mcDESPOT should be immune to this effect, assuming the analysis includes and accurately estimates the myelin water residence time parameter that is intended to account for exchange. However, omitting exchange from the mcDESPOT model so it is not accounted for, as with GRASE, does not remove the discrepancy between MWF and f_M_ values^25^.

The caveat that the residence time must be accurately estimated becomes problematic in the context of recent studies evaluating the mcDESPOT analysis. Degeneracy of parameter solutions in mcDESPOT, a fundamental problem afflicting many MRI microstructure mapping techniques^49^, was first shown by Lankford and Does studying mcDESPOT precision with Cramér‐Rao lower bounds^12^. Imprecision of mcDESPOT solutions has been further demonstrated by Zhang et al. and Bouhrara et al., who both showed flat residual plots of mcDESPOT parameter fits^14,25^. This can result in substantial instability in metric values, especially for moderate or low signal-to-noise data^14^. They also showed that the instability could be reduced by removing fit parameters, specifically by ignoring the effects of exchange^25^.

A recent investigation by West et al. further showed that mcDESPOT solutions are inaccurate and imprecise when exchange is included in the model^15^. As in previous work, they found that ignoring exchange improved the stability of the analysis, but at the cost of significantly biased results^15^. West et al. also found that changes in mcDESPOT analysis results were non-linear with respect to changes in their true values, and that changing a single parameter could induce marked changes in other, static parameters^15^. For example, a change in the T_1_ or T_2_ value of intra-/extra-cellular water can result in a large change in f_M_ results, even when the true value of f_M_ has not changed^15^. The notable sensitivity of f_M_ to pathological tissue differences could be in part explained by certain pathology-related changes to T_1_ or T_2_ inducing a large, non-linear response in the f_M_ results^15^.

West et al. suggested that unpredictable bias in mcDESPOT results can be at least partially mitigated by the use of consistent acquisition and analysis parameters^15^. However, our results suggest that even with consistent acquisition and analysis conditions, changes in signal characteristics due to the presence of pathology (multiple sclerosis) or unique tissue structures (spinal cord) propagate into f_M_ values in a way that does not consistently reflect MWF values.

The anatomical structure of the spinal cord likely plays an important role in our observed breakdown of the MWF-f_M_ relationship as well as the lack of contrast observed in our f_M_ spinal cord results. The non-linear behaviour of the mcDESPOT analysis procedure could lead to biased results that do not necessarily reflect the true underlying values. Spinal cord WM is similar to that of corpus callosum in the brain, and cord GM is similar to putamen^50^. Therefore, based on contrast between brain values, we can expect that MWF will have more contrast between these regions than f_M_. Another consideration is the presence of a low f_M_ boundary around the cord, larger than that visible for MWF, which is likely related to partial volume effects with cerebrospinal fluid. Partial volume effects could indicate imperfections in the motion correction alignment of each image volume in mcDESPOT data sets. Misalignment from motion relates to the fact that mcDESPOT acquires central k-space data separately for each image volume, spread across the entire acquisition time, whereas GRASE acquires central k-space data only once. Imperfect alignment of mcDESPOT image volumes would lead to blurred metric maps and reduced contrast between ROIs inside the cord.

MWF and f_M_ can be used to identify pathology on an individual basis in the brain or spinal cord^51–53^. Here, we demonstrated this using Z score maps, shown in Fig. 3, which showed greater spatial extent and magnitude of severity for mcDESPOT f_M_ Z scores, compared to those of GRASE MWF.

The high sensitivity of mcDESPOT to pathological differences has been previously demonstrated in the brain by O’Muircheartaigh et al.^26^. Although there is no definitive explanation, the high sensitivity of mcDESPOT is likely due to multiple compounding factors. Relatively low inter-participant f_M_ variability, as evidenced by the low values of f_M_ standard deviation between participants (Fig. 2), will tend to amplify Z score values. Another influence may be reduced specificity to myelin, which would grant f_M_ sensitivity to a variety of pathological changes compared to MWF. Strong correlation (median ρ = 0.8) between f_M_ and quantitative T_1_ results from mcDESPOT^26^ provides evidence suggesting that f_M_ is closely related to, if not driven by, relaxation values. Considering this, f_M_ may not provide enough additional independent, myelin-specific information, compared to the quantitative T_1_ metric from DESPOT1-HIFI^20^, to justify the increased acquisition time to acquire a full mcDESPOT data set.

Despite their different acquisitions, analyses, and confounding factors, MWF and f_M_ Z score histogram plots in Fig. 3 show similarity between participants and anatomical structures. Similarity between Z scores and the higher sensitivity of f_M_ to pathological tissue changes support the interpretation that MWF and f_M_ are both sensitive to myelin content but that f_M_ has reduced specificity due the unpredictable, non-linear analysis response to signal changes. For specificity to myelin, recent technical developments allow acquisition of multi-echo T_2_ relaxation data with compressed sensing acceleration, which can provide improved spatial and parametric resolution in less acquisition time, compared to the GRASE sequence presented here^54^. When possible within time constraints, studies should ideally include multiple myelin-sensitive metrics. Additional metrics incorporated in a multi-modal imaging approach can provide supplemental information as well as increase confidence in the findings.

In conclusion, we have produced and provided normative reference MWF and f_M_ atlases for both brain and cervical spinal cord. In comparing these atlases, we found that MWF and f_M_ follow an approximately linear relationship for regions with MWF <  ~ 10%, above which the relationship breaks down and f_M_ no longer increases in tandem with MWF. In the context of demyelinating disease pathology, Z score maps from f_M_ showed greater spatial extent and magnitude of severity. The high sensitivity of f_M_ is a benefit for detecting pathological changes but is likely related to the mcDESPOT analysis procedure propagating MR signal changes into the f_M_ results non-linearly and greatly limits the ability to attribute those differences solely to changes in myelin. Despite the fundamental differences in these two techniques, MWF and f_M_ Z score maps showed overlapping areas of low Z score and similar trends between patients and brain and spinal cord regions. Ultimately, these results will guide future choice of myelin-sensitive quantitative MRI and help improve retrospective interpretation of clinical studies using multi-echo T_2_ relaxation or steady-state myelin imaging measures in the context of healthy or diseased central nervous system.

## Methods

### Ethics

The study operated under ethics approval from the University of British Columbia Clinical Research Ethics Board. All participants provided written informed consent. All methods were performed in accordance with the relevant guidelines and regulations.

### Participants

Data were collected from 28 healthy participants without a history of brain or spinal cord disease or injury (10 male and 18 female, mean age 46 years, range 22–65 years). To facilitate comparison of MWF and f_M_ in the context of pathology, data were also collected from two participants with clinically definite multiple sclerosis fulfilling the 2017 revised MacDonald criteria for diagnosis^55^: one participant with RRMS (female, age 26 years, expanded disability status scale (EDSS^56^) 1.5, disease duration 3 years) and one with PPMS (female, age 64 years, EDSS 8.5, disease duration 29 years).

### MRI

Imaging was performed at the UBC MRI Research Centre on a 3.0T MRI scanner (Achieva, Philips Medical Systems, Best, The Netherlands).

Brain MRI were acquired using an 8-channel head coil.

A sagittal 3D T_1_-weighted magnetization-prepared rapid gradient-echo image (T_1_w) was acquired with TR = 8.1 ms, echo time (TE) = 3.5 ms, inversion time (TI) = 1052 ms, shot interval = 3000 ms, FA = 8°, resolution = 1.0 × 1.0 × 1.0 mm^3^, field of view (FOV) = 256 × 256 × 165 mm^3^, acquisition time = 6 min 26 s.

Multi-echo T_2_ relaxation data were acquired using a 3D multi-echo GRASE sequence with TR = 1073 ms, TE = 8 ms, echo spacing (ΔTE) = 8 ms, 48 echoes, acquired resolution = 1.0 × 2.0 × 5.0 mm^3^, reconstructed resolution = 1.0 × 1.0x2.5 mm^3^, FOV = 230 × 190 × 100 mm^3^, slice oversampling factor = 1.3, sensitivity encoding (SENSE^57^) acceleration factor = 2, and acquisition time = 7 min 31 s.

Steady-state mcDESPOT brain acquisitions consisted of multiple SPGR, a single IRSPGR, and multiple bSSFP images, all acquired with a common resolution = 1.7 × 1.7 × 1.7 mm^3^ and FOV = 217 × 217 × 156 mm^3^. SPGR were acquired with TR = 6.5 ms, TE = 3.6 ms, and FA = 2, 3, 4, 6, 9, 13, and 18°; IRSPGR with TR = 6.5 ms, TE = 3.2 ms, TI = 450 ms, and FA = 5°; and bSSFP with TR = 5.8 ms, TE = 2.9 ms, FA = 7, 11, 15, 19, 24, 30, and 47° and phase cycling patterns of 0° and 180° for each FA. Total mcDESPOT acquisition time, for all FA and phase cycling patterns, was 9 min 26 s.

Cervical spinal cord imaging used a 6-channel spine coil, with all imaging FOV centred at the C2/C3 level of the cervical spinal cord and angulation aligned with the cord.

An axial 2D multi-slice T_2_*-weighted multi-echo gradient echo image (T_2_*w) was acquired with TR = 815 ms, TE = 6.5 ms, ΔTE = 8.2 ms, 5 echoes (cumulated on the scanner), FA = 28°, acquired resolution = 0.8 × 0.8 × 2.5 mm^3^, reconstructed resolution = 0.3 × 0.3 × 2.5 mm^3^, number of slices = 16, slice gap = 0.25 mm, FOV = 150 × 150 × 44 mm^3^, and acquisition time = 5 min 7 s.

Multi-echo T_2_ relaxation MWI data were acquired as adapted by Ljungberg et al.^27^ using a 3D multi-echo GRASE sequence with TR = 1501 ms, TE = 10 ms, ΔTE = 10 ms, 32 echoes, acquired resolution = 0.75 × 0.75 × 5.0 mm^3^, reconstructed resolution = 0.63 × 0.63 × 2.5 mm^3^, FOV = 180 × 150x40 mm^3^, slice oversampling factor = 1.2, SENSE acceleration factor = 2, and acquisition time = 8 min 36 s.

Steady-state mcDESPOT cord acquisitions had a common acquired resolution = 0.75 × 0.75 × 4.00 mm^3^, reconstructed resolution = 0.70 × 0.70 × 4.00 mm^3^, and FOV = 180 × 201 × 56 mm^3^. SPGR were acquired with TR = 5.5 ms, TE = 2.7 ms, and FA = 2, 3, 4, 6, 8, 10, 13, and 18°; IRSPGR with TR = 5.5 ms, TE = 2.7 ms, TI = 350 ms, and FA = 5°; and bSSFP with TR = 10.0 ms, TE = 5.0 ms, FA = 7, 14, 21, 28, 35, 42, and 49° and phase cycling patterns of 0° and 90° for each FA. Total mcDESPOT acquisition time, for all FA and phase cycling patterns, was 6 min 7 s.

The T_1_w brain and T_2_*w cord images were acquired for their strong white/gray matter contrast, to facilitate alignment of anatomical structures between participants. All acquisitions were performed without cardiac or respiratory triggering. Brain and cord acquisitions for GRASE and mcDESPOT differed slightly due to differences in optimisation of parameters for each application^8,27^.

### Quantitative MRI analysis

GRASE data was analyzed using a temporally-regularized non-negative least squares algorithm with stimulated echo correction (https://mriresearch.med.ubc.ca/news-projects/myelin-water-fraction/), 40 T_2_ relaxation delta functions logarithmically spaced from 15 to 2000 ms, 8 refocusing flip angles between 90 and 180°, and a χ^2^ regularization factor of 1.02. The MWF was calculated as the fraction of total signal with 15 < T_2_ < 40 ms.

mcDESPOT data underwent motion correction to align the imaging volumes before the DESPOT1-HIFI code was used to obtain an estimated FA correction map from the SPGR and IRSPGR data^20^. Using the FA correction map, mcDESPOT analysis was performed to obtain the f_M_. The analysis was matched to that of a previous study on mcDESPOT stability, using a 3-pool model and the same stochastic-region-contraction parameter boundaries: f_M_ [0.00000001,0.35], myelin water T_1_ [300,650] ms, myelin water T_2_ [1,30] ms, free water T_1_ [700,T_1,max_] ms, free water T_2_ [50,165] ms, and myelin water residence time τ [25–600] ms.

### Image analysis and template creation

**Figure 4:**
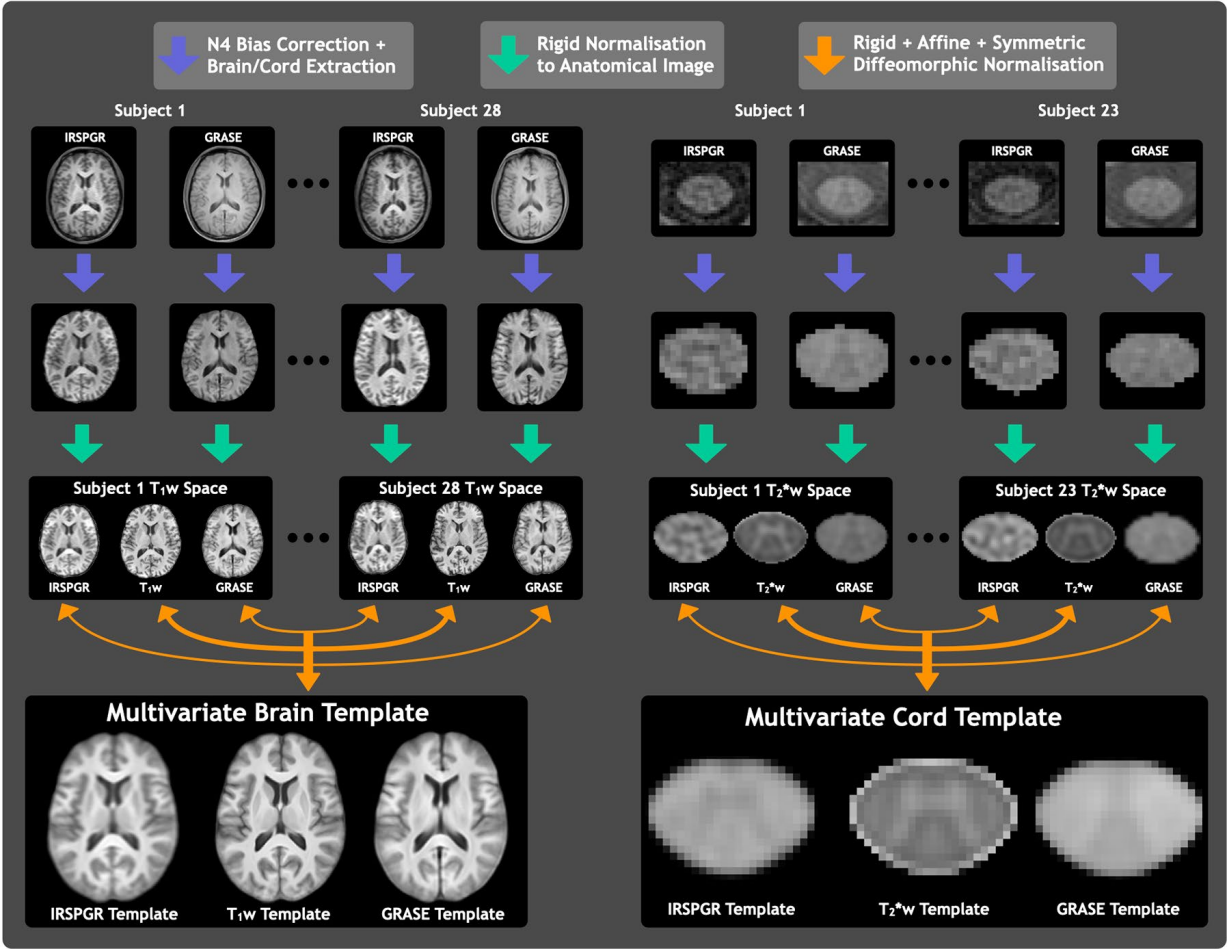
Workflow depicting the multivariate template creation pipeline for brain and cervical spinal cord data. Image registrations used the GRAdient-echo and Spin-Echo (GRASE) echo 1 image from multi-echo T_2_ relaxation data, and the inversion-recovery-prepared spoiled gradient recalled echo (IRSPGR) image from the steady-state multi-component Driven-Equilibrium Single-Pulse Observation of T_1_ and T_2_ (mcDESPOT) data.

Advanced Normalization Tools software (ANTs), Spinal Cord Toolbox (SCT), and some FMRIB Software Library (FSL) tools were used for image analysis^58–60^. Non-linear registrations used symmetric diffeomorphic normalization (SyN) transformations. To prevent introduction of circularity bias, no quantitative maps were involved in image registrations^61^. Workflow for the multivariate template creation process is outlined in Fig. 4.

All T_1_w, GRASE echo 1, and IRSPGR brain images underwent N4 bias field correction for low frequency intensity nonuniformities^62^. Brain segmentations were initialized by registration with the OASIS template and priors^63^ then refined using Atropos *n*-tissue segmentation^64^. Each participant’s GRASE echo 1 and IRSPGR images were masked to brain tissue before being rigidly aligned with their corresponding T_1_w image and masked again using the higher-resolution T_1_w brain segmentation.

For spinal cord images, cord segmentations were generated for each image modality using T_2_*w, GRASE echo 16, and IRSPGR images with an automated tool (sct_propseg^65,66^) before quality control was performed manually. The cord segmentations were used to rigidly align GRASE echo 1 and IRSPGR with T_2_*w cord images. Finally, all images were masked using the higher-resolution T_2_*w cord segmentation.

Multivariate template creation was performed by iteratively co-registering the aligned and masked GRASE echo 1, IRSPGR and anatomical images (T_1_w for brain, T_2_*w for cord) using iterative rigid, affine, and SyN transformations. Registrations were guided by mutual information (for linear stages) and neighborhood cross-correlation (for SyN) similarity metrics. The T_1_w and T_2_*w similarity metrics were weighted twice as heavily as those of GRASE and IRSPGR, to account for their better resolution and tissue contrast.

### Region of interest definition

A reference anatomical T_1_-weighted template was used to generate brain ROIs. This reference template was created in-house using data from 100 healthy volunteers, including those in the current study. After registration between our T_1_w template and the reference T_1_w template, JHU-ICBM-DTI-81 WM labels and MNI structural regions, optimized for the reference template using the probabilistic joint label fusion framework^67^, were transformed to our multivariate template space. Atropos *n*-tissue segmentation was performed on our T_1_w template to provide WM and GM segmentations^64^.

To generate spinal cord ROIs, registration was performed between our T_2_*w spinal cord template and the T_2_*-weighted SCT PAM50 template^68^, centred at the C2/C3 level of the cord, and refined using a manually defined cord GM segmentation to improve alignment of intra-cord structure. This provided probabilistic cord ROIs, which were thresholded and binarized to only include voxels with probability > 0.5, to reduce partial volume effects.

### Multiple sclerosis image analysis

RRMS and PPMS images were processed the same as those of healthy participants, except they were excluded from template creation. Instead, their T_1_w and T_2_*w images were registered to the T_1_w and T_2_*w templates, and transformations were concatenated to align their MWF and f_M_ maps in template space for comparison. Since the goal of the study was not to derive clinical conclusions, images from participants with multiple sclerosis were not segmented into lesion/non-lesion tissue.

### Statistical analysis

From the healthy participant MWF maps, aligned in template space, atlases were created by calculating the voxel-wise mean and SD between participants, then cropped to exclude regions not covered by all individual participant MWF and f_M_ FOV. For the mean MWF and f_M_ atlases, the mean and SD within each ROI were calculated. Mean MWF and f_M_ ROI values were compared across all healthy control participants using a paired, two-tailed t test. MWF and f_M_ Z score maps were calculated by subtracting the mean atlas from the MS participant quantitative map, then dividing by the SD atlas. Z score maps were also masked to exclude highly variable voxels, where the coefficient of variation (calculated voxel-wise as the SD divided by the mean) was over 0.75.

## Data Availability

Ethics approval did not allow for making individual subjects’ imaging data available. However, the anatomical templates, quantitative atlases, and ROIs created and analysed in this study have been made openly available^33^ along with example code for the template creation process^69^.
